# Improvement of cereal‐ and legume‐derived protein quality with selenium and sulfur for plant food production

**DOI:** 10.1002/jsfa.14061

**Published:** 2024-12-12

**Authors:** Muna Ali Abdalla, Munshi Mohammad Sumon, Karl Hermann Mühling

**Affiliations:** ^1^ Institute of Plant Nutrition and Soil Science, Kiel University Kiel Germany

**Keywords:** Se, S, globulin family, lupin, wheat flour, meat alternatives, baking quality

## Abstract

Selenium (Se) is essential for human and animal health and nutritional status. As humans cannot produce Se, it must be obtained from the diet. Adequate Se supplementation improves innate immunity, increases antioxidant capacity and helps prevent various disorders. Sulfur (S) is an indispensable nutrient that affects plant growth, performance and yield. Cereals and legumes are global staple foods, and their proteins are considered sustainable plant‐based meat alternatives, which are increasingly popular. Owing to their physicochemical similarities, the crosstalk between Se and S influences cereal and grain‐legume derived proteins. Modifications induced by Se and S might improve the protein quality of harvested cereal and legume grains. This timely review not only identifies the knowledge gaps in this research area focusing on Se and S enrichment in cereals and legumes but also emphasizes the potential of this unexplored area for new applications. S enrichment resulted in better quality properties in the bread of wheat flour and stimulated the expression of S‐rich globulins and albumins in legumes. Se supplementation enhanced the emulsifying capacity of legumes (e.g. chickpeas). The improvement of protein fractions in cereal and legume grains has the potential to revolutionize protein processing to offer new alternatives to produce an array of S‐ and Se‐enriched cereal and legume products. © 2024 The Author(s). *Journal of the Science of Food and Agriculture* published by John Wiley & Sons Ltd on behalf of Society of Chemical Industry.

## INTRODUCTION

Sulfur (S) and selenium (Se) are essential elements that occur in proteins, being constituents of the amino acids cysteine (Cys), methionine (Met), selenocysteine (SeCys) and selenomethionine (SeMet). A previous study highlighted the exceptional nature of these amino acids.^1^ Their redox activity in response to physiological circumstances gives rise to various posttranslational protein modifications, and redox signaling.^1^ At physiological pH, the Cys residue is found as a thiolate anion (Cys–S^−^); consequently, it will be directly oxidized by H_2_O_2_ to synthesize the sulfenic residue (Cys–SOH). This leads to allosteric modifications that can modulate protein activity. The sulfenic residue, once formed, can be reduced to a thiolate anion by the disulfide reductases, thioredoxin and glutaredoxin to restore the protein function to its native state.^2^

Se is a metalloid owing to its location between the metals and the nonmetals in the periodic table. This unique position makes selenoproteins serendipitous candidates with catalytic activity in redox transformations and signaling pathways. The strong redox activity of S‐ and Se‐containing amino acids (e.g. Cys, Met, SeCys and SeMet) has attracted the interest of chemists and biological chemists, inspiring further research on their potential role in protein functions.

### Contrasting characteristics of selenium and sulfur

Se and S are classified as chalcogens in the periodic table, which means that they have similar ionic radii, covalent radii and chemical properties.^3^ Hence, they share assimilatory pathways in plants. Due to its greater atomic radius and lower electronegativity than S, Se is a better nucleophile than S, making Se's attacks on electrophiles more potent.^4^ The dissociation of the Se—H bond has a significant influence owing to the differences between Se and S, where Se—H bond dissociation is easier than that of the S—H bond, resulting in greater SeCys acidity than Cys. Therefore, the possibility exists that Se—H bonds in a peptide chain can be more readily oxidized compared with the corresponding S‐containing peptides. Accordingly, Se‐containing peptides may have a diverse range of applications involving selective chemical modifications, which can lead to additional functional groups, including disulfide crosslinks, which substantially increase the stability of protein structures.^5^

This report seeks to understand the importance of the Se and S interplay in the synthesis of cereal‐ and legume‐derived proteins. It sheds light on a new approach to producing improved proteins in terms of their quality and techno‐functional properties. This strategy might facilitate the discovery of new Se‐ and S‐enriched meat‐substitute applications, by benefiting from the properties of cereal‐derived proteins (e.g. wheat, maize, rice and oats), which can be used to provide the necessary structural and textural characteristics to plant‐based meat alternatives. In addition, legume proteins (such as soybean, lupine, lentils, chickpeas and peas) are considered novel candidates in the development of meat analogs due to their emulsification, gelation and foaming properties.

### Cereal and legume proteins make them attractive candidates in the food industry

Cereal and legume grains are the main dietary sources of food, feed and fuel globally. Legume seeds are among the richest food sources of proteins and amino acids for human and animal nutrition.^6^, ^7^ Additionally, cereals and legumes are highlighted as valuable sources of healthy plant proteins and environmentally sustainable food sources as part of the transition to healthier diets. Besides the role of protein content in dough strength in breadmaking, whole wheat flour provides significant nutritional ingredients, such as vitamins, dietary fiber and essential minerals.^8^ According to the protocol of Osborne (1907), based on their solubility, proteins are divided into different groups by solvents, including water‐soluble albumins, salt‐soluble globulins, alcohol‐soluble prolamins and dilute acid‐ and alkali‐soluble glutelins.^9^ Protein fractions of various cereal and legume crops are listed in Tables 1 and 2. Gluten proteins are mainly responsible for the unique baking properties of wheat flour. When flour and water are mixed, they create a viscoelastic cohesive dough that can be kneaded. This can only be produced from wheat flour. After washing the kneaded dough with water, and removing starch and other ingredients, the resulting isolated residue is called gluten, which is responsible for the plasticity and stability of the dough.^10^ Gluten proteins consist of gliadins, which are responsible for the extensibility and viscosity of dough, as well as glutenins, which contribute to the elastic and strength properties of dough.^11^ Gliadins are divided into three groups: *α*/*β*‐, *γ*‐ and *ω*‐gliadins. Glutenins are classified into low‐molecular‐weight (LMW) and high‐molecular‐weight (HMW) glutenin subunits (GS). HMW glutenins contribute to dough strength and extensibility due to their intramolecular and intermolecular disulfide bonds, while LMW glutenins only form intermolecular disulfide bonds with other glutenin proteins and are more abundant than HMW glutenins. Among these proteins, the influence of LMW glutenins on the properties of bread baking in terms of quality is like that of gliadins, contributing to the viscosity of the viscoelastic characteristics of the dough.^12^, ^13^

**Table 1:** Protein fractions of cereal grains and their importance in food processing^18^, ^19^

Cereal crop	Osborne fraction				Importance of protein fraction in food industry
	Albumin	Globulin	Prolamin	Glutelin	
Wheat	Leucosin	Edestin	Gliadin	Glutenin	Gluten is crucial during the breadmaking process. Glutenin gives elasticity to the dough, and gliadin contributes to its extensibility
Corn			Zein	Zeinin	Zein has potential polymerization properties that facilitate the production of membranes, polymeric films and coating materials, making it a possible replacement for synthetic polymers. The industrial applications of zein include the pharmaceutical, food, fiber, cosmetics and adhesives industries^1^, ^20^
Rye			Secalin	Secalinin	Rye proteins are not capable of forming a dough structure. However, they are essential for building the strong taste and flavor of rye sourdough bread. Protein hydrolysis, followed by peptide or amino acid metabolism formed through enzymatic degradation facilitated by lactic acid bacteria, is crucial to produce aroma precursors. The Ehrlich pathway constitutes the major degradation pathway of amino acids during the fermentation of sourdough^9^
Oats			Avenin	Aveninin	The oat‐specific prolamin avenin has lower celiac toxicity compared with prolamins from other grains. Accordingly, oat‐based, gluten‐free products, which are not contaminated with gluten from other cereal grains, are essential in a gluten‐free diet. However, gluten‐free oat products are currently extensively contaminated^21^
Barley			Hordein	Hordeinin	The main storage protein in barley is hordein, which accounts for 30–50% of the total seed protein.^22^ In addition, its concentration influences the quality of baked and brewed products^23^

**Table 2:** Crude protein content and protein fractions in some legumes (adapted from Montoya *et al.* ^29^)

Legume	Crude protein (g kg^−1^ DM)	Osborn fractions, % of total protein				Ref.
		Albumin	Globulin	Prolamin	Glutelin	
Soybean	300–500	10	85–95	—	—	30
White lupin	310–350	10–20	80–90	—	—	31
Common bean	213–313	12–30	54–79	2–4	20–30	32
Pea	212–329	21	66	—	12	27
Faba bean	229–385	20	65	—	15	27
Cowpea	209–346	45	51	1	3	33

Consumption of oats is becoming a popular way of achieving a healthy diet. Besides their high nutritional quality, oats contain a gluten‐like prolamin protein called avenin, which is a safer component in most cases for celiac patients.^14^

In legumes, globulins constitute about 70% of the seed protein. They are classified as legumins (11S), vicilins (7S) and convicilins (in lower amounts). Legumins and vicilins constitute the major globulins/storage proteins in legume crops.^6^ Moreover, prolamins and glutelins are found in meagre quantities.^15^ Being rich in essential amino acids complementary to cereal crops as well as being considered gluten‐free, legumes are safe for people with gluten intolerance.^16^ Legume proteins, including bean and pea proteins, have been reported to improve the overall quality of gluten‐free muffins and produce edible films and coatings.^17^

### Protein quality measurements in cereals and legumes and the resulting techno‐functional properties for potential food applications

Protein quality encompasses the composition of amino acids, as well as the digestibility and absorption of food peptides and their constituent amino acids following digestion. These factors identify a protein's ability to cover the body's metabolic demand and should be considered when evaluating the environmental impact of food proteins.^24^, ^25^ *In vivo* protein quality measurement includes protein efficiency ratio, in addition to protein digestibility‐corrected amino acid score, which determines protein quality by taking into account human amino acid needs and digestibility. Additionally, the digestible indispensable amino acid score is recognized as a potential approach for assessing the protein quality of foods. Moreover, *in vitro* protein assays are receiving increased attention due to ethical issues and lower costs.^26^

The techno‐functional characteristics of cereal proteins, including the higher water‐holding and oil‐absorption capacities, are excellent properties that make them promising candidates in food systems.^27^ Additionally, besides their higher protein content, leguminous proteins demonstrated interesting technological properties such as foaming capacity, emulsification and gelation, in addition to their digestibility. As nutrient‐dense crops, together with their high sustainability, affordability, consumer acceptability, satisfactoriness and palatability, leguminous proteins are ideal as substitutes for animal‐derived proteins in the food industry and applications.^28^

In addition to legume‐protein‐based, novel foods, such as meat alternatives, extruded products and healthy protein snacks,^34^ cereal proteins have been used to produce films with higher‐quality physical characteristics.^35^ Furthermore, packaging industries have developed legume‐protein‐based films and coatings.^36^

A previous study evaluated the potential of techno‐functional characteristics of legume‐ and cereal‐derived proteins of some varieties cultivated in Canada. It was found that soybean, green pea and hull‐less barley flours revealed greater water hydration capacities, which qualify them as attractive ingredients in binding applications such as manufactured meat products. Moreover, red lentils and hull‐less barley flours showed better foaming capacities, making them good candidates for egg substitutions. Additionally, cereal flours exhibited lower foam stability and emulsifying activity than legume flours, which qualifies them as thickening agents for soups and sauces.^37^

## SELENIUM AND SULFUR ENRICHMENT OF CEREALS AND LEGUMES HAS THE POTENTIAL TO OFFER SEVERAL BENEFITS TO THE FOOD INDUSTRY VIA IMPROVING PROTEIN QUALITY

**Figure 1:**
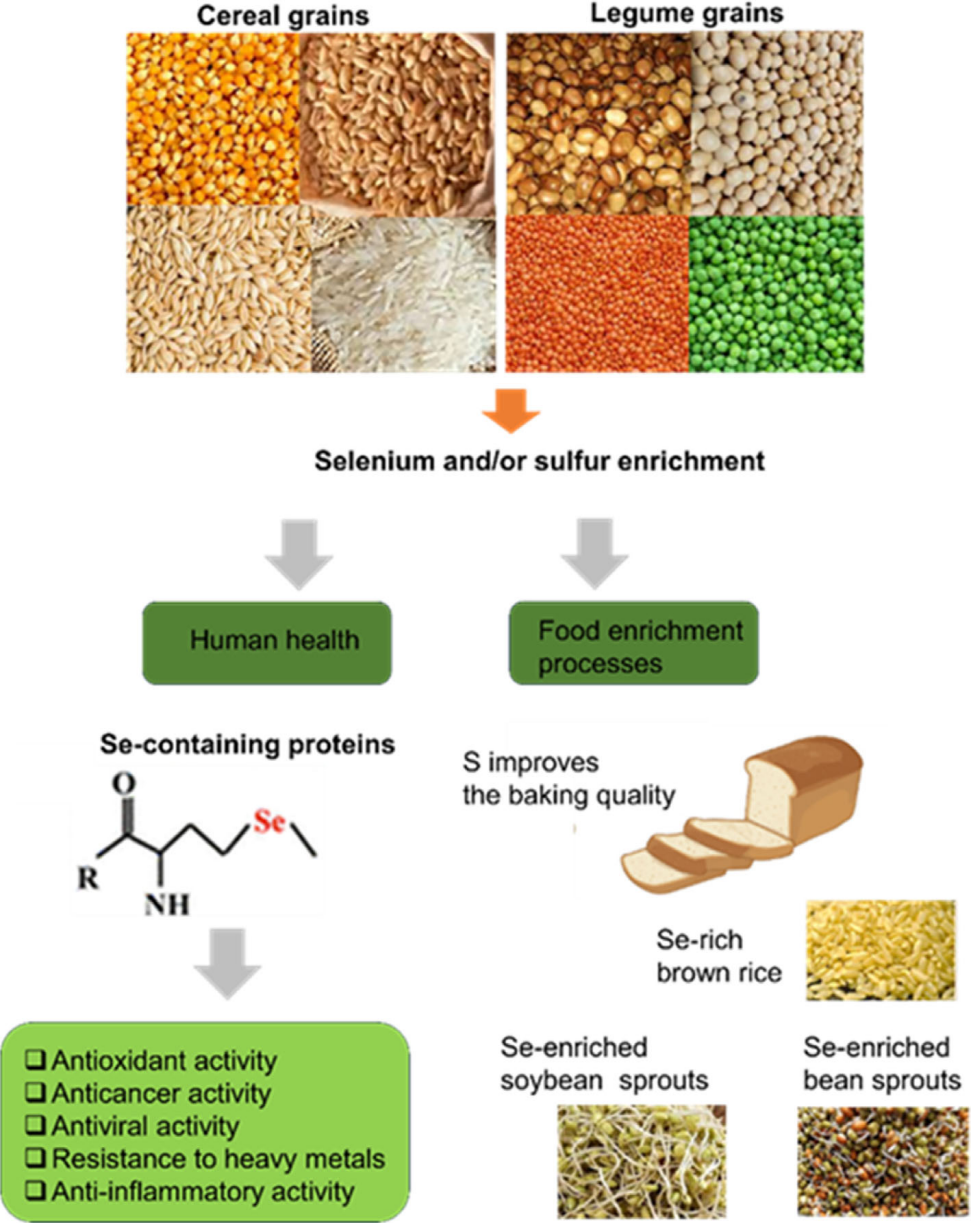
Se and S applications impact the protein content of cereal and legume grains, enhancing their nutritional quality and human health.

Se assimilation in plants can impact metabolic pathways of both S and nitrogen (N); alterations in S assimilation under Se application can efficiently affect N metabolism in terms of amino acid biosynthesis and proteins.^38^ The optimal dose of Se can increase the nutritional value of food crops by enhancing the total protein and amino acid levels,^39^ making selenoproteins much better than Se supplementation alone. In a previous report by Ekiz *et al*. investigating Se biotransformation in *Ganoderma lucidum*—a well‐known mushroom that was used in Chinese traditional medicine to strengthen the immune system and promote overall health status—the authors believed that inorganic Se was transformed into water‐soluble selenoproteins.^40^ Accordingly, its hydroxyl and superoxide anion radical scavenging capacity was three times more than that of the original proteins, and its antioxidant activity was closely related to Se levels. The proteome analysis of untreated and Se‐treated apple leaves was performed using two‐dimensional electrophoresis and mass spectrometry characterization. In addition, new protein spots were detected in the apple proteome of the Se‐treated leaves. Furthermore, their functions included binding calcium ions, increasing the stability of photosystem II, strongly delaying RuBisCO degradation, improving the efficiency of electronic transfer and implementing energy transformation.^41^ Se application may lead to the synthesis of selenoproteins (i.e. glutathione peroxidases and thioredoxin reductases).^42^ Plant‐derived Se‐containing proteins/peptides containing the rare amino acid Cys are involved in various stress signaling pathways and play significant roles in cellular defense mechanisms. In addition, they provide remarkable health benefits beyond the dietary requirements of Se. Moreover, selenoproteins have a wide spectrum of pleiotropic effects including antioxidant, anticancer and anti‐inflammatory activities. Additionally, selenoprotein P has a crucial role in improving cognition in patients with heart failure, in addition to its important role in Alzheimer's disease and type 2 diabetes (Fig. 1).^43^, ^44^

Based on research data gathered using proteomic approaches, S or Se supplementation to cereal and legume grains demonstrated positive changes in protein composition and quality (Fig. 1). For instance, S application favorably enhanced S levels in wheat grains and dough extensibility, consequently improving overall baking volumes.^45^, ^46^, ^47^ In a previous research, it was shown that applying S significantly increased the amount of S‐rich proteins, while decreasing S‐poor proteins. Late S treatment altered the analysis of the proteome. Afterwards, a small‐scale baking test was used to assess the baking quality of whole‐grain flour. The authors discovered that late S application had a positive impact on the composition of gluten proteins and baking quality.^46^ Moreover, it has been reported that the proportion of S‐rich and S‐poor hordeins is affected by N and S limitations.^48^, ^49^ Legumes are known for their lower S‐containing amino acid concentrations and tryptophan (Trp), while they are an excellent source of lysine (Lys).^50^

S supply increased seed protein in various legumes, including globulins in soybean and lupine,^51^, ^52^ and globulins and albumins in pea.^53^, ^54^ Higher S induces the expression of S‐rich globulins and albumins, while S deprivation stimulates the expression of S‐poor globulins.^55^

Se agronomic biofortification of wheat has previously been used in Australia as an alternative to breeding to enhance grain Se concentration.^56^ A recent study showed that Se foliar application produced Se‐enriched soybeans without altering the protein structure and function.^57^ Moreover, a previous report indicated that Se treatment in the form of selenite during germination of brown rice was transformed to Se‐containing proteins.^58^ However, the distribution of Se in food crop proteins is not yet fully understood.

Evidence from several studies suggests that the enrichment of both S and Se can improve the protein quality of cereal and legume grains, as documented below.

### Sulfur efficiently influences protein patterns in cereal and legume grains

S is a macronutrient that is crucial for plant growth, development and physiological processes. It is the fourth most significant element in crop plants, after N, P and K,^59^ which have a significant effect on crop productivity. Plants require 0.1–0.5% S on a dry matter basis^60^; hence it is essential in the production of sulfolipids, iron–sulfur clusters (Fe–S), lipids, polysaccharides, vitamins (e.g. thiamine and biotin), disulfides, peptides (e.g. phytochelatins and glutathione) and cofactors (e.g. *S*‐adenosylmethionine and CoA).^61^ S constitutes a structural element of plant protein disulfide bridges,^62^ particularly in cereals and legumes. Several studies have demonstrated that S fertilization can positively impact the quality of proteins in cereal grains.^63^, ^64^ Some examples of the influence of S on cereal and legume protein and quality are listed in Table 3. Cai *et al*. investigated the influence of Cys and inorganic S when applied during two growth stages on the storage protein and the baking quality of wheat. At the jointing stage, fertilization was more effective in improving storage protein than a basal application, because S could be used more for the S metabolism of grains when supplied at the late stage. However, most of the S applied before sowing was used for the metabolism of the whole plant, leading to a decrease in its amount due to early soil application and the availability of less S for the grain‐filling stage. Consequently, among the two forms of S, Cys application was the best treatment as it improved not only the yield of grain, percentage of glutenin, GMP, LMW‐GS and S content but also baking quality. This is because, in this form, S utilization and absorption in wheat were high and S‐rich protein, mostly glutenin, was significantly accumulated.^65^ Overall, the effect of S application on the production of HMW‐GS, LMW‐GS and GMP polymerization was primarily mediated by the regulation of Cys. Tao *et al*. studied the influence of S supply and short‐term high‐temperature stress (HTS) on the components of wheat flour proteins and agreed with Cai *et al*. that S supply significantly enhanced the volume of bread and, consequently, the baking quality of wheat flour. In addition, the authors found that HTS increased total protein levels along with reducing starch synthesis. It also hastened maturity, shortened the grain‐filling period and decreased grain yield and total starch accumulation. However, the application of S mitigated the damaging impact of HTS on wheat yield and flour quality. HTS decreased nitrate reductase activity in flag leaves by accelerating plant senescence during the later life stage of the grain‐filling period. Subsequently, the plants did not ameliorate the adverse effects. Thus, the activity of nitrate reductase in flag leaves was diminished. This could be attributed to the reduction of NO_3_^−^ to NH_4_^+^, which consequently decreased. Furthermore, the loss of available NH_4_^+^, a substrate of the glutamine synthetase reaction pathway, can cause a decrease in glutamine synthetase activity and starch synthesis and a significant increase in grain protein content. The activity of nitrate reductase and glutamine synthetase was elevated by S application, resulting in starch synthesis. The content of Cys induced gluten formation and improved its elasticity, thereby increasing processing quality of the wheat. Met promoted wheat's nutritional quality, which was enhanced by applying S fertilization.^66^ That study highlighted the significance of S in alleviating environmental stress, such as heat stress, to improve wheat yield and the protein quality of wheat flour.

**Table 3:** Previous examples of the impact of S on cereal and legume protein and further processing

Studied crop	Treatments and conditions	Findings	Ref.
Wheat	Inorganic S and Cys were applied as the basal dose and at the jointing stage	Cys application exhibited a remarkable impact on bread quality characteristics, including reduction in the hardness of bread (up to 69.3%). The chewiness of bread was the highest, and the volume was enhanced by 109.8%	65
Wheat	Plants grew in soil with two doses of S (0 and 14 mg kg^−1^) and three doses of Se (0, 1 and 2 mg kg^−1^) under glasshouse conditions	The grain of wheat contains Se, which is mainly present in organic forms. Spitfire cultivar grain has 97% SeCys, while Longsword cultivar grain has 70% SeMeCys. In contrast, sulfate fertilization has an antagonistic influence on the assimilation of Se(VI) into reduced forms (e.g. SeCys and SeMeCys)	75
Maize	N was used at rates of 0, 3.2, 12.9 and 25.8 CO(NH_2_)_2_ g pot^−1^. S was used at rates of 0, 7.7, 23.1 and 86.2 MgSO_4_ g pot^−1^	N and S fertilizers increased the amount of storage protein in maize grain. However, applying excess N led to decreased essential amino acids (EAA), whereas more S enhanced EAA in the protein. More importantly, the treatment combination of 0.24 g N kg^−1^ (moderate) and 0.24 g S kg^−1^ (high) in soil showed the highest percentage of EAA and protein in maize grain	69
Barley	Six field experiments were performed using three levels of N (0, 100, and 160 kg ha^−1^) and two S levels (0 and 10 kg S ha^−1^)	S application affected hordein composition, enhanced malt extract, apparent attenuation limit and diminished malt hardness; grain hordein and N levels remained unaffected	70
Winter wheat	A 2‐year experiment in which N and S rates were 56, 101, 146 kg N ha^−1^, and 0 and 22 kg S ha^−1^, respectively	S increased the ratio of polymeric to monomeric protein concentration. An elevated polymeric protein content was associated with firmer dough and better bread quality	67
Winter wheat	S application and exposure to short‐term HTS at 20 days postanthesis	Applying S improved total protein and fractions, including albumin, gliadin, glutenin and globulin content and overall wheat flour quality	66
Cowpea	Four levels of Se and S were used in two consecutive years	High Se with moderate S reduced the free amino acid concentrations, and the percentage of storage protein varied widely due to the S and Se crosstalk	73
Navy bean	S was applied at 17, 26, 35 and 44 kg ha^−1^	The study found that adding S fertilization at different rates did not show any effect on navy bean protein level, protein quality or functionality	74
Soybean	Agrochemicals, pasty, powdery and solutes with nanostructured S were used as a source of S	Among the agrochemicals, solute and powdery forms enhanced soybean protein fractions	71
Soybean	S sources, namely gypsite, elemental S powder and elemental S pastille, along with five rates, 0, 50, 100, 150 and 200 mg kg^−1^, were applied	Elemental S pastille is not recommended as an S source because the low S content in the leaf decreases the yield. Elemental S pastille and elemental S powder particle sizes were crucial for S availability in soybeans. Deficiency of S was observed at all gypsum levels, leading to diminished globulin protein synthesis and an elevated albumin fraction. However, gypsum, gypsite and elemental S powder induced the analyzed storage protein fractions	72

The impact of S and N on protein levels and quality, dough rheology and asparagine accumulation in winter wheat was investigated.^67^ A higher asparagine level reacts with reducing sugars to form acrylamide in baked products, which leads to adverse effects on human health. Wilson *et al*. found that S supplementation diminished asparagine levels in a year‐long experiment. Moreover, evaluation of the dough rheology showed that S treatment enhanced the average farinograph stability (from 9.2 to 14.6 min). The study revealed the significant role of S supply for the qualities of protein and wheat flour in winter wheat production, especially in S‐deficient soils. Concerning the ability of S to affect acrylamide formation, Stockmann *et al*. supported the previous study that S could positively reduce acrylamide accumulation, especially under S‐limitation conditions. At the same time, no effect was observed in soils with adequate S levels.^68^ As supported by previous authors, the combined treatment with S and N demonstrated increased protein and essential amino acids. The ratio of essential to nonessential amino acids was also enhanced to provide balance. The presence of two important amino acids, lys and trp, indicated that the protein quality of maize was also significantly enhanced. N and S fertilizers synergistically increased the protein content and the balance of amino acids. The study reported that applying N and S produced significantly high protein concentrations in maize grains.^69^ The interplay of N and S and the resultant effect on protein characteristics (e.g. gluten) in wheat has been evaluated. An optimum ratio of N and S is necessary for establishing gluten (especially S‐rich gluten), and consequently baking, quality. Elevated N combined with lower S negatively altered gluten protein composition, resulting in losses of grain quality.^47^ Concomitantly, the interaction of S and N on the hordein fraction quantity, malt extract and quality in barley has been studied.^70^ Although both elements affected hordein fractions, S application positively influenced malt quality (malt extract and fermentation potential, and diminished malt hardness) and composition of hordeins. The impact of S on malt extract might be attributed to its effect on other malt characteristics that define the quality of extract (e.g. enhancing the activity of amylolytic enzymes).^70^

The influence of S‐containing agrochemicals on soybean growth and protein accumulation has also been studied. The agrochemicals in various forms were found to have a positive impact on soybean protein content, increasing all fractions of the reserve protein compared to the control.^71^ Likewise, the effect of various S sources (including elemental S pastille, gypsum, gypsite and elemental S powder) on the yield and storage protein in soybeans was assessed. S deficiency was found at all concentrations for elemental S pastille, leading to lower globulin percentages and a large amount of albumin and glutelin in soybean seeds. This might be attributed to the insufficient S supply required for the growth and development of soybean plants, resulting in reduced yields. Moreover, protein fractions were enhanced in response to S sources, for example, gypsum, gypsite and elemental S powder. Additionally, the granulometry of elemental S pastille and elemental S powder sources was beneficial for S fertilization in soybean plants.^72^

Silva *et al*. described the reason for low Cys levels in cowpeas when treated with high Se and moderate S: the concentration of reactive oxygen species (ROS) within the cell that leads to lipid peroxidation may increase when high Se levels are present. This is a stressful condition arising due to increased Se, which may impair the synthesis of free amino acids, as Cys is known to be a glutathione precursor, which is an essential component of ROS scavenging. Consequently, at these high Se levels, available Cys is changed into glutathione to act in its defensive role in the scavenging of ROS.^73^ The role of S in the synthesis of Met and Cys and the possible replacement of Se in these amino acids confirm that both elements can affect protein levels. Deushi *et al*. found that increasing the level of S application did not influence the protein concentration in the flours of navy beans. Three essential amino acids, namely Met, Cys and Trp, are deficient in navy beans, and S fertilization cannot increase their availability. The apparent reason for the minimum S effect on navy bean quality is attributed to environmental conditions that reduce the response of plants to S availability.^74^

### Selenium enriches cereal and legume proteins

Various prospective investigations in humans have confirmed the close association between blood Se concentration and the risk of disease with possible adverse effects occurring at both high and low levels of the optimal range required for the biological functions of some or all selenoproteins.^76^ Elevated serum Se levels are associated with a higher risk of the prevalence of diabetes mellitus. Insufficient Se intake (<20 μg per day) has been correlated to a number of serious health problems including Alzheimer's or Parkinson's disease, cancer, male infertility, thyroidal dysfunctions, heart disease, cognitive loss and myodegenerative diseases.^77^, ^78^, ^79^ Se enrichment of food crops can enhance Se levels in the latter, leading to better Se dietary intake. Accordingly, Se deficiency can be addressed with Se biofortification.^80^ In plants, a Se concentration more than approximately 5 mg kg^−1^ is considered toxic; however, plant tolerance in terms of toxicity varies. Excess Se in plants causes general symptoms, such as withering, chlorosis and stunted shoot growth.^81^ Moreover, its toxicity negatively affects proteomic and nonproteomic processes (e.g. changing redox status and lipid peroxidation). Such damage includes malformed selenoproteins and oxidized/nitrated proteins due to stress.^82^ Plants can substitute S with Se due to their similar properties. Accordingly, selenate (SeO_4_^2−^) follows the same pathway as SO_4_^2−^ in translocation and assimilation,^83^ and this process substitutes Cys and Met with SeCys and SeMet in protein incorporation.^84^

Table 4 presents several examples of Se enrichment and its effect on cereal and legume proteins. For instance, Kaur and Sharma investigated Se‐induced changes in protein‐bound Se and the storage protein accumulation of wheat grain in the reproductive stage. Those authors found that Se application reduced numerous protein fractions, namely albumins, globulins, prolamins and glutelin, as well as total soluble protein. Among the protein fractions, glutelin contained the highest amount of Se, and prolamin had the second highest, followed by albumins and globulins. A deeper analysis of the polypeptide bands and the structure of the protein revealed that an increase in Se doses affected polypeptide bands while increasing the flexibility and structural unfolding of the protein. Overall, protein or amino acids with excess Se binding may cause harmful effects, as they may exchange the sulfhydryl group from normal protein. Hence, a minimum dose should be maintained to avoid toxicity in the Se enrichment process.^85^ Accordingly, the minimum dose should be maintained to avoid toxicity in the Se enrichment process.^86^

**Table 4:** Previous examples of the influence of Se on cereal and legume protein (during the last 10 years)

Studied crop	Treatments and conditions	Findings	Ref.
Wheat	The experiment was designed to use two Se sources (selenate and selenite, 2 and 4 mg Se kg^−1^ soil)	When wheat was exposed to Se, it led to the accumulation of large proteins that had unfolded and flexible secondary structures. Although the overall levels of protein and storage proteins in Se‐enriched plants decreased, a higher amount of protein‐bound Se was observed in wheat grains grown on seleniferous soils. This can be advantageous for Se fortification purposes	85
Colored‐grain wheat	Foliar Se fertilization was performed at early grain filling using varied Se concentrations (37.5–112.5 g ha^−1^)	The concentration of gliadin and glutenin significantly increased, while both albumin and globulin decreased with increasing Se concentration	88
Wheat	The experiment consisted of eight treatments with different growth stages (preflowering and prefilling stage). Foliar rates of Se were 20 and 100 g ha^−1^ (as selenite and selenate)	Applying selenite or selenate to the leaves of wheat at prefilling rather than at preflowering significantly enhanced the Se levels in wheat grains. SeMet is the primary Se form in grains, accounting for approximately 67–86% of total Se	87
Wheat, rice and maize	Samples were collected at Se‐rich sites from three different locations in each field	All grains, except rice, prolamins, and glutelin, showed significantly higher levels of Se than other proteins. However, in rice, the Se level was higher in globulin than in glutelin. The Se concentration of proteins was found to be directly correlated with their bioaccessibility	93
Rice	This was a field experiment with five Se rates (0, 10, 25, 50 and 100 g ha^−1^) applied as selenate via soil	Se improved protein quality	90
Rice	Two genotypes were used (high and low Se uptake), named (Z3057B) and (Chenghui727), respectively	The LMW protein (13.4 kDa) bound more Se and accounted for the greatest total protein amount (40.76%)	92
Soybean	Seeds were soaked in varied Se solution levels (0, 5, 30 and 60 mg L^−1^ Na_2_SeO_3_) for 6 h	Detection and quantification of Se species, mainly SeMet and MeSeCys, in germinated soybeans and different levels of enriched Se found in soybean proteins, mostly 7S and 11S. Amino acids, except Metin 11S, remained unchanged under Se treatment. In addition, considering the protein structure, only *α*‐helix contents decreased with enhanced Se concentration	94
Soybean	Seeds were soaked using different Se levels (0, 5, 10, 20, 40 and 60 mg L^−1^)	Within 24 h of germination, inorganic Se was converted into an organic form, with 89.3% of the total Se bound to soybean protein	95
Soybean	Foliar selenite (SeO_3_) or selenate (SeO_4_) were applied at rates of 60 g ha^−1^	Most Se in soybean seeds is in the form of SeMet (>90%) after foliar spray of different Se sources. However, soybean seeds had higher Se levels and recovery efficiency after selenate treatment than after selenite treatment. Se accumulated in SPI and 11S at greater levels in all Se‐enriched soybean proteins. The stability and functionality of the protein structure remained unaffected by Se, indicating no significant impact. Finally, a foliar spray of selenate was favorable for Se‐enriched soybeans, and SPI and 11S were found to be efficient protein carriers of Se	57
Mung bean	Foliar application using varied Se levels content of 0, 15, 30 and 45 g ha^−1^	Se treatment of 30 g ha^−1^ significantly enhanced Se, protein and fat content	89
Chickpea	Chickpea seeds were germinated for 4 days after being soaked with Se (0, 1 or 2 mg (100 g)^−1^ seeds)	Elevated Se level in the glutelin protein fraction was found in germinated plants	96
Chickpea	Seeds were germinated for 48 h using 96 mg L^−1^ of selenite	Se enhanced the emulsifying properties of albumins and glutelin	97

Se foliar application was performed using two Se sources supplied at several stages of wheat growth. Selenate application accumulated more Se than selenite in wheat grain, and the prefilling stage of wheat was considered the optimum time for selenate application via foliar spray. Importantly, Se translocation may be faster in the grain at the reproduction stage. Although the authors did not evaluate the protein quality of Se‐enriched wheat grains, it is essential to enhance the Se amount in grains, which not only increases human Se consumption but might also affect Se‐bound protein and consequently impact protein quality. Therefore, the authors recommended that applying selenate to the leaves at a rate of 20 g ha^−1^ during the prefilling stage is beneficial for increasing Se accumulation in wheat grains.^87^ Moreover, Se foliar application was found to be effective in enhancing protein quality and inducing micronutrient accumulation, including iron and zinc.^88^ In particular, it enhanced gliadin and glutenin and diminished albumin and globulin‐colored grain wheat. Yet, the gluten quality remained undisturbed due to Se application. Se foliar spraying demonstrated a remarkable effect on the production of primary metabolites (e.g. amino acids and their derivatives) and bioactive secondary metabolites (e.g. phenolic acids and flavonoids). Among these key metabolites required for the synthesis of proteins, *N*‐methyl‐l‐glutamate, l‐saccharopine and *S*‐allyl‐l‐cysteine were upregulated.^89^ The study of Reis *et al*. provided valuable insights into the protein quality of rice. Those authors found that Se enrichment increased Se levels in leaves and grains and storage proteins in rice grains. The quantities of albumin, globulin, prolamin and glutelin were enhanced, supporting the positive effect of Se on N metabolism.^90^ It is very important to mention that Se accumulation in grains favored the interaction between Se and the various protein fractions (albumin, globulin, prolamin and glutelin). Additionally, owing to the nonspecific Se utilization in plant proteins, it can be assumed that the amino acids, which are mainly present in all fractions, such as Cys, Met, glutamic acid, arginine, aspartic acid and lysine, are facilitating the Se association with proteins. They include S‐containing or charged group amino acids, which determine the binding affinity toward transition metal ions and nonmetals.^91^ In a recent study, the effects of Se on the protein level and distribution in various parts of rice have been investigated. The Se protein was detected with a molecular weight of 13.6–122.6 kDa. Furthermore, each protein with a specific molecular weight was found to be bound with Se. However, the strength of the binding with Se is inversely related to the molecular weight of the protein. As a result, approximately 67.5% of the total Se is bound to proteins with a molecular weight lower than 38.8 kDa.^92^ The important finding of Zeng *et al*. is that there is a link between protein structures of varying molecular weights and Se binding strength. This might be beneficial for further study for evaluating the quality of different protein fractions of Se‐enriched rice, which might be helpful for rice applications in food processing. Dhanjal *et al*. cultivated three cereal crops located in a seleniferous belt to observe Se distribution in wheat, rice and maize grains. Se content of cereals in their storage proteins fraction was distributed in ascending order, that is, wheat > maize > rice, where prolamin had the highest amount of Se than other protein fractions. Those authors also studied the gastric simulation and digestibility of gastrointestine during intestinal digestion. The authors found better Se accessibility to variations between protein fractions and types of cereal.^93^ The findings enhanced knowledge of the impact of Se on cereal grain protein and correlated with Se bioaccessibility. These grains with high Se content can potentially be used as supplements to meet the nutritional requirements of individuals in Se‐deficient areas.

Among Se application methods, seed soaking before germination can be recommended as a useful process to obtain Se‐enriched sprouts. For instance, Huang *et al*. found that Se species, including MeSeCys and SeMet, were the predominant species in soybean sprouts treated with Se. Se application did not alter protein levels compared with the control group. However, the accumulation of total and essential amino acids was higher under a lower Se supply and diminished in response to greater Se application.^95^ Huang *et al*. examined two soybean proteins, 7S and 11S, from raw soybeans, germinated soybeans and germinated soybeans biofortified with Se, along with sprout powder. They found that GS‐Se had a significantly higher Se concentration than germinated soybeans, with total Se ranges of 41.5–80.5% and 19.5–21.2% in SeMet and MetSeCys, respectively. Met obtained from 11S showed a minor effect of Se treatment. Moreover, a negative correlation was observed between the increasing Se content and the percentage of the *α*‐helix, while no significant changes were found with the other structures. Moreover, Se enrichment enhanced antioxidant activity in 7S.^94^ These findings point to the potential to produce Se‐enriched germinated soybean protein, which can be beneficial in improving Se‐enriched plant‐based meat analogs. Another study supported the potential of Se‐enriched soybean protein for food manufacturing, in which the authors investigated Se uptake and Se species distribution in different soybean proteins, namely soybean protein concentrate, soybean protein isolate (SPI), glycinin (7S) and *β*‐conglycinin (11S). Among the Se species, SeMet was more than 90%, which explains why it is considered the supreme organic type of Se. In addition, the Se content in SPI and glycinin was higher than that in soybean protein concentrates and *β*‐conglycinin in seeds treated with Se.^57^ It is worth mentioning that the beneficial effect of Se on soybeans was observed without altering the protein structure and function by Se; however, the processing of soybean proteins using different techniques may change the structure and function of proteins compared to natural ones.^98^

Se supplementation of cereals and legumes not only improved protein quality but also enhanced the production of beneficial secondary metabolites, including dihydroquercetin, anthocyanins, catechins and flavone derivatives in wheat,^99^ isoflavonoids in germinated soybeans^94^ and several isoflavonoids in addition to two pterocarpan phytoalexins in chickpea sprouts.^100^

The techno‐functional emulsifying properties of Se‐enriched proteins of germinated chickpeas were also explored. Protein fractions exhibited greater emulsifying characteristics. Moreover, the greater Se‐enriched glutelin protein fraction exhibited elevated cellular antioxidant properties along with the highest emulsifying activity.^97^ This is an outstanding application that can pave the way for the use of Se‐enriched glutelin emulsions in food manufacturing.

Bread supplementation with sprouted Se‐enriched chickpea sourdoughs has also been investigated.^101^ A remarkable effect of Se‐enriched chickpea sourdough in producing organic acid during the fermentation process was observed, along with the promotion of lactic acid bacteria during the stages of sourdough and dough formation. Moreover, besides their high nutritional value, Se‐enriched chickpea breads are able to cover the Se dietary intake of humans. Likewise, Se‐enriched sprouted chickpea flour was used to supplement wheat flour for breadmaking.^102^ The fortified bread exhibited greater protein quality and adequate organoleptic characteristics.

Although Se enrichment has been demonstrated as an efficient strategy, a comprehensive evaluation of the different biofortification techniques is crucial to guarantee the safety of Se‐rich legumes and cereal products.

## SUMMARY AND OUTLOOK

**Figure 2:**
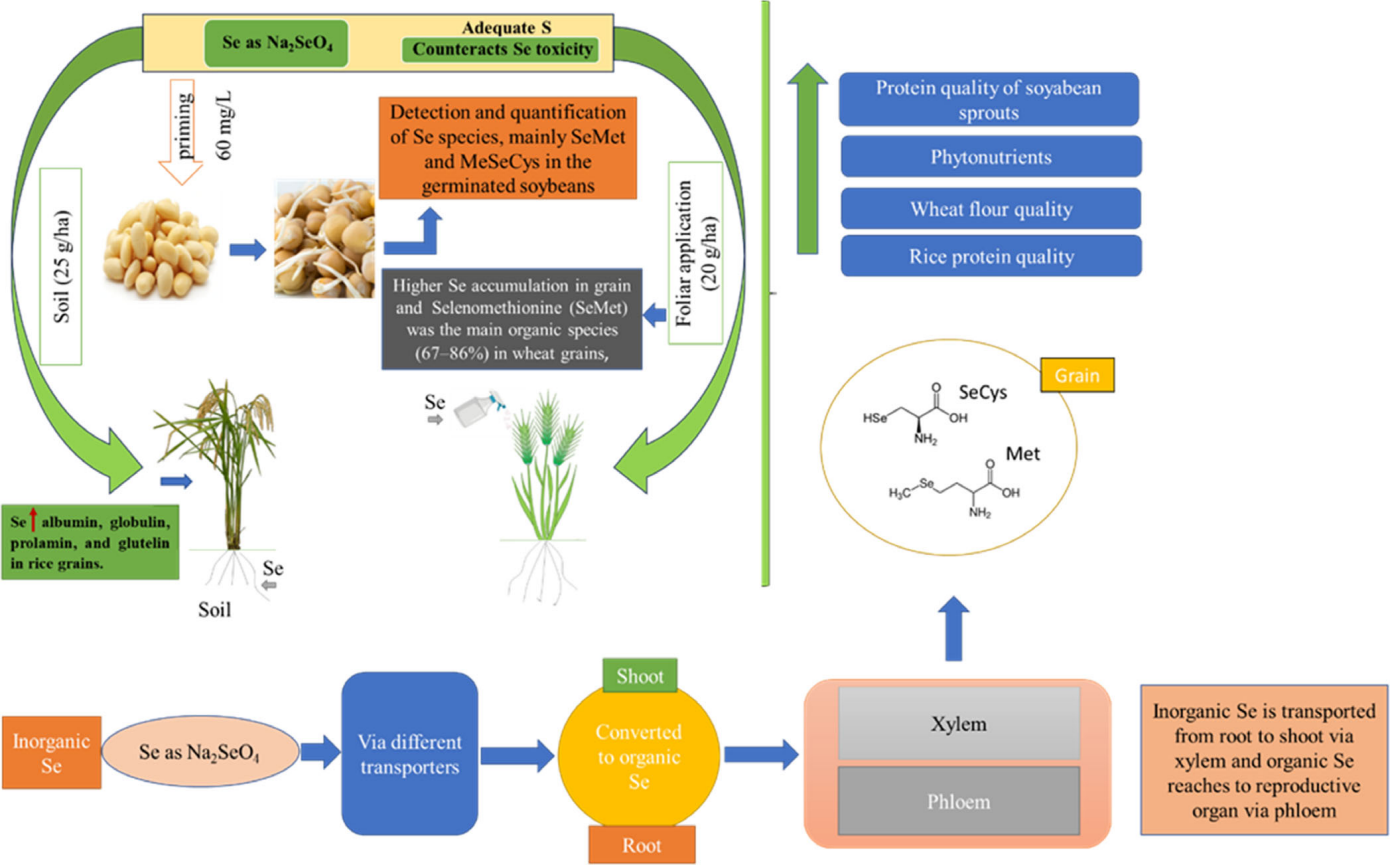
Approaches of Se application, including seed priming, soil fertilization and foliar application in the presence of sufficient S, and the uptake, translocation and accumulation of Se through the xylem and phloem to the grain.

This review provides insight into the current status of the influence of Se and S treatments on protein quality and techno‐functional properties in cereals and legumes. Cereal proteins can impact the structure and quality of baked products, further impacting dough properties by improving gluten proteins. Legume grains are a great replacement for meat; therefore, they are known as a source of vegetarian protein and play a crucial role in food formulation and processing. In addition, owing to the functional properties of their proteins, including emulsification, gelation, and foaming, they may be appropriate substitutes for some of the present dietary animal source proteins.^103^ Although the industry for plant‐based meat alternatives is growing to satisfy the strongly growing consumer preferences for meat substitutes, the functional properties of legume grain proteins do not yet meet the needs of modern food manufacturing, which is becoming more interested in high‐quality legume seed protein properties. Accordingly, research on improving the functional properties of protein modification is required. For instance, lupin has a high protein content but poor emulsifying characteristics in comparison with other legume proteins.^104^ A previous study confirmed that S fertilization improved protein quality, enhancing its degradable fraction. Moreover, lupin oil quality was ameliorated by producing higher oleic and linolenic acid contents and lower amounts of erucic acid.^105^ S application enhanced the S‐containing amino acids in some lupin varieties; however, it diminished lysine and threonine levels without altering the protein concentration.^106^ Further, a recent investigation based on a three‐year field experiment revealed that increasing S dose significantly enhanced the total and true protein content and their respective S ratios in seeds of narrow‐leafed lupin.^107^ Silva *et al*. showed that the interplay between Se and S may positively affect cowpea storage protein and protein concentration.^73^ Interestingly, S sources, such as GY, GI and ESPO, enhance all storage protein fractions in soybeans.^72^ The impact of Se and S enrichment on grain protein, especially pulses, needs to be investigated for a better understanding of their effect on protein structure and pattern. Three approaches to Se application are described in Fig. 2: seed priming, soil fertilization and foliar application. All the approaches are very effective, according to which crops are cultivated and the objective of cultivation. Many studies have demonstrated that foliar application is the best approach for Se biofortification. There are very narrow ranges of Se doses; among them, 20–40 g ha^−1^ showed effective uptake and translocation of Se in plants.^87^, ^88^, ^108^ Se shares physicochemical properties with S, and a low level of Se can stimulate S uptake. In addition, high Se toxicity can be mitigated by S application. Se amino acids may be incorporated into proteins, altering their structural folding and disrupting their function,^109^ which largely depends on the ratio of Se to S in the plant rather than solely on the Se level.^110^ In addition, the toxicity resulting from the incorrect insertion of Se amino acids into proteins can be eliminated by the methylation of SeCys and SeMet to form methyl‐SeCys or methyl‐SeMet, respectively, which can be used to synthesize DMSe as in non‐Se‐hyperaccumulators and DMDSe in Se‐hyperaccumulators.^109^, ^111^

**Figure 3:**
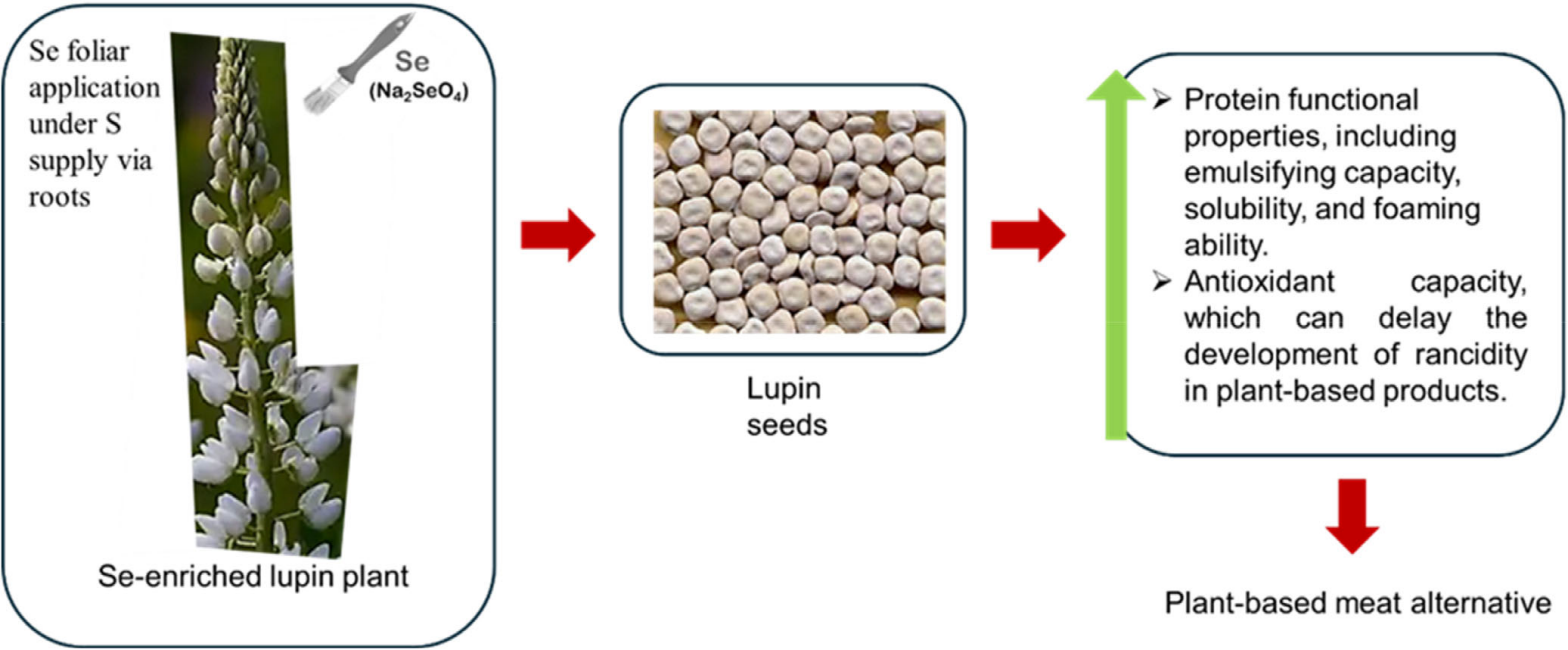
A Proposed effect of Se biofortification on the protein functional properties of lupin as an example of the richest legume protein.

Via soil application, Se enrichment has been noted to enhance the amount of Se bound to various storage protein fractions in wheat leading to an elevated level of HMW proteins bearing unfolded and flexible secondary structures.^85^ A novel approach can investigate how Se under S application can affect important proteins in wheat flour, such as globulins, *α*‐ and *γ*‐gliadins and LMW‐GS, which may impact breadmaking quality. Hence, Se enrichment in soybeans demonstrated effects on the dominant proteins 7S and 11S, in addition to the techno‐functional characteristics and antioxidant activity.^94^ In the presence of sufficient S, Se application is assumed to improve the protein quality of lupin by its significant effect on amino acid balance, techno‐functional properties and digestibility. Moreover, Se induced antioxidant capacity in lupin sprouts as reported by a previous study^112^ (Fig. 3). Hence, a combination of Se and S will be a better approach for providing not only protein quality for the food industry but also a source of Se needed for human health.

## CONCLUDING REMARKS

Se biofortification has proven to be a successful method for enhancing Se content in various food crops, which is beneficial for human health. However, in‐depth research focused on the influence of Se on the functional properties of the proteins of important crops such as cereals and legumes is urgently needed, especially concerning S nutrition. The accumulation of Se in different protein fractions of Se‐enriched cereals and legumes has shown significant differences, which could be attributed to the amino acid composition in these fractions. Interestingly, the presence of S‐containing amino acids (Cys and Met) in the amino acid composition can make the interaction of both elements (S and Se) in different protein fractions more efficient, especially in terms of Se accumulation. Additionally, the quality of protein‐bound Se should be evaluated, as well as the techno‐functional properties of cereal and legume crops. These crops are ideal plant‐based protein sources. This approach can help create cereal‐ and legume‐protein‐rich food, which can enhance their extensive use in food manufacturing to address the growing popularity of plant‐based meat alternatives.

One of the most challenging aspects of this approach is detecting and quantifying Se‐containing proteins in different protein fractions, owing to their complex and varied nature and substantial molecular weights. Hence, to support this strategy, an urgent requirement for standardized protocols and databases seems to exist. Currently, appropriately monitoring and discovering the technological processes involved in Se‐enriched legumes and cereals for the production of novel foods is necessary.

## AUTHOR CONTRIBUTIONS

MAA and KHM conceived the topic. MAA wrote the manuscript. MAA, MMS and KHM contributed to the final version of the manuscript. MAA, MMS and KHM substantially revised it. All authors have read and agreed to the published version of the manuscript.

## CONFLICT OF INTEREST

The authors have no conflicts of interest to declare relevant to this article's content.

## CONSENT FOR PUBLICATION

All authors have approved the final draft of this manuscript for submission and have given consent for the publication of identifiable details.

## Data Availability

The data that support the findings of this study are available on request from the corresponding author. The data are not publicly available due to privacy or ethical restrictions.
